# The influence of patient case mix on public health area statistics for cancer stage at diagnosis: a cross-sectional study

**DOI:** 10.1093/eurpub/ckz024

**Published:** 2019-03-14

**Authors:** Matthew E Barclay, Gary A Abel, Lucy Elliss-Brookes, David C Greenberg, Georgios Lyratzopoulos

**Affiliations:** 1 The Healthcare Improvement Studies (THIS) Institute, University of Cambridge, Cambridge, UK; 2 Department of Public Health and Primary Care, University of Cambridge, Cambridge, UK; 3 Medical School (Primary Care), University of Exeter, Exeter,UK; 4 National Cancer Registration and Analysis Service, Public Health England, London, UK; 5 Epidemiology of Cancer Healthcare and Outcomes (ECHO) Research Group, Department of Behavioural Science and Health, University College London, London, UK

## Abstract

**Background:**

Summary statistics comparing the stage at diagnosis of geographically defined populations of cancer patients are increasingly used in public reporting to monitor geographical inequalities but may be confounded by patient case mix. We explore the impact of case-mix adjustment on a publicly reported measure of early stage at diagnosis in England.

**Methods:**

We analyzed data used for publicly reported statistics about the stage of patients diagnosed with 1 of 11 solid tumours in 2015 in England, including information on cancer site (bladder, breast, colon, rectum, kidney, lung, melanoma, non-Hodgkin lymphoma, ovarian, prostate, endometrial), age, gender, income deprivation and population-based commissioning organization. We investigated how cancer site and other patient characteristics influence organizational comparisons and attainment of early-stage targets (≥60% of all cases diagnosed in TNM stages I–II).

**Results:**

Adjusting for patient case mix reduced between-organization variance by more than 50%, resulting in appreciable discordance in organizational ranks (Kendall’s tau = 0.53), with 18% (37/207) of organizations being reclassified as meeting/failing the early-stage target due to case mix.

**Conclusion:**

Summary statistics on stage of cancer diagnosis for geographical populations currently used as public health surveillance tools to monitor organizational inequalities need to account for patient sociodemographic characteristics and cancer site case mix.

## Introduction

Across the world, the increasing disease burden associated with cancer is motivating new approaches to public health surveillance to support cancer prevention and control. Monitoring of stage at diagnosis for cancer patients of organizations responsible for the planning and administration of local healthcare systems (including screening programmes and diagnostic services) may be a promising approach. In England, substantial recent improvements in cancer registration have enabled the publication of annual statistics for such geographically defined populations corresponding to commissioning organizations (known as Clinical Commissioning Groups or CCGs). These summarize, for 11 cancer sites combined, the proportion of patients who are diagnosed at early stage.^1^ CCGs typically have a population of about 250 000 residents and a varied sociodemographic profile. As cancer incidence varies by sociodemographic group, and certain cancer sites (e.g. lung and breast cancer) have both contrasting sociodemographic predictors and stage distribution,^2^ variation in patient case mix may confound such statistics. Previous evidence raises concerns about chance variation and potential bias from missing data for early-stage indicators, but the potential influence of patient case mix poses additional concerns.^3^ We therefore examined the degree by which patient characteristics and cancer site affect observed variation in cancer stage at diagnosis between English CCG populations.

## Methods

### Dataset

We analyzed data on all patients aged 30–99 diagnosed in England in 2015 with cancer of the colon (ICD10 C18), rectum (C19–C20), lung (C33–C34), melanoma (C43), female breast (C50), endometrial (C54), ovarian (C56–C574), prostate (C61), renal (C64) and bladder cancer (C67) and non-Hodgkin’s lymphoma (NHL, C82–C85), the same sites used in the English ‘early stage at diagnosis’ indicator. These data included information on site of diagnosed tumour, gender, age (5-year age group), Index of Multiple Deprivation 2015 income deprivation quintile,^4^ stage at diagnosis and CCG of residence. We classified stage at diagnosis as ‘early’ (TNM stages I–II) and ‘late’ (TNM stage III–IV)—consistent with the definition of the ‘early stage at diagnosis’ indicator in current use.

Stage data were highly complete (91%, see Results), and given empirical evidence supporting the appropriateness of complete case analysis when comparing stage at diagnosis between CCGs,^3^ we included only patients with valid stage in all analyses.

### Statistical analysis

Initially, we assessed how case-mix adjustment (CMA) affected the distribution of early stage at diagnosis between CCGs using multilevel logistic models, including CCG as a random effect. We compared the size of the CCG random effect between models with and without fixed effect case-mix variables. We used the user-written programme *meresc* to rescale results for fair comparisons between these models.^5–7^

We subsequently considered five possible specifications of an ‘early stage at diagnosis’ indicator. Starting from a specification without adjustment for case mix (i.e. emulating the convention in current use), we incrementally considered case-mix factors in order of their impact on between-CCG variance when used as single adjustors. Cancer site had the largest impact when used as a single adjustor (decreasing the degree of variance), followed by deprivation quintile, while gender had minimal impact; adjusting for age at diagnosis increased the between-CCG variance. Therefore, the five possible specifications of the indicators comprised:

1. No CMA
2. Adjustment for cancer site only
3. Adjustment for cancer site and income deprivation quintile
4. Adjustment for cancer site, income deprivation quintile and gender
5. Adjustment for cancer site, income deprivation quintile, gender and age group

We calculated the case-mix-adjusted CCG percentage of tumours diagnosed at early stage (TNM stages I–II). We fit fixed-effects logistic models to the whole dataset and recycled the model estimates to predict the stage distribution for each CCG that would have been seen if the case mix in each CCG was the same as the whole national population of those diagnosed with these cancers in that year.^8^ We assessed the impact of CMA on observed (crude) CCG percentage of early stage at diagnosis by investigating:

1. Change in the 5th–95th percentile of the CCG range of early-stage proportion.
2. The Kendall’s tau correlation between crude and case-mix-adjusted organizational ranks in respect of the percentage of patients diagnosed at an early stage. Kendall’s tau belongs to the broader family of statistical measures used to examine correlation and is particularly apt for examining concordance of organizational ranks in the context of CMA.^9^^,^^10^ It measures the concordance of all possible pairwise comparisons.^11^ A value of 1 indicates perfect agreement, −1 indicates complete disagreement and 0 indicates half of comparisons were concordant and half discordant.
3. Changes in organizational classification according to the pay-for-performance target threshold (whether 60% or greater percentage of cancer patients in a CCG are diagnosed at early stage).

We summarised results graphically by producing paired coordinate plots showing adjusted CCG proportion early stage and adjusted rank on the early-stage indicator under each of the five CMA specifications we considered.

All analyses were performed in Stata v13.1.^12^

## Results

Our dataset included 208 586 patients diagnosed in 2015, distributed across 207 geographically defined CCG populations in England (table 1), of whom 189 632 (91%) with known stage were included in our analysis (see Supplementary table A1 for breakdown of stage categories by cancer site). There were substantial between-area differences in proportions of patients diagnosed with specific cancer sites. For example, in the 5% of CCGs with the fewest lung cancer patients less than one in eight patients (<12%) had lung cancer, compared with more than a quarter of patients (>26%) in the 5% of areas where lung cancer was most common (table 1). There was also substantial between-area variation in the age and deprivation distribution of people diagnosed with cancer (table 1).

**Table 1:** Descriptive statistics on overall sample of staged diagnoses of colon, rectal, lung, breast, endometrial, ovarian, prostate, renal, bladder cancer, melanoma and non-Hodgkin’s lymphoma in English residents in 2015, with univariate test for differences between Clinical Commissioning Groups (CCGs)

Factor	National		5th percentile of CCGs	Median CCG	95th percentile of CCGs	*P*
Total diagnoses (of staged tumours)						
	189 632	100%	375.4	771	2161.80	
Proportion early stage (of staged tumours)						<0.001
	108 750	57%	51%	57%	62%	
Stage at diagnosis						<0.001
1	65 497	35%	26%	31%	36%	
2	43 253	23%	17%	21%	24%	
3	35 331	19%	14%	17%	21%	
4	45 551	24%	18%	22%	27%	
Cancer site (ICD10)						<0.001
Colon (C18)	20 561	11%	8%	11%	13%	
Rectal (C19–C20)	10 145	5%	4%	5%	7%	
Lung (C33–C34)	34 419	18%	13%	18%	26%	
Melanoma (C43)	11 796	6%	3%	6%	9%	
Female breast (C50)	41 697	22%	17%	22%	28%	
Endometrial (C54)	6781	4%	2%	4%	5%	
Ovarian (C56–C574)	4176	2%	1%	2%	3%	
Prostate (C61)	35 653	19%	14%	18%	25%	
Renal (C64)	7578	4%	3%	4%	6%	
Bladder (C67)	7271	4%	3%	4%	5%	
Non-Hodgkin’s lymphoma (C82–C85)	9555	5%	4%	5%	7%	
Age at diagnosis						<0.001
30–39	3599	2%	1%	2%	4%	
40–44	4215	2%	1%	2%	4%	
45–49	8103	4%	3%	4%	6%	
50–54	12 106	6%	4%	6%	9%	
55–59	15 651	8%	6%	8%	11%	
60–64	21 308	11%	9%	11%	13%	
65–69	31 594	17%	13%	17%	20%	
70–74	28 822	15%	12%	15%	18%	
75–79	27 032	14%	11%	14%	17%	
80–84	20 326	11%	8%	11%	13%	
85–89	11 957	6%	4%	6%	8%	
90–99	4919	3%	1%	3%	4%	
Gender						<0.001
Men	92 977	49%	44%	49%	55%	
Women	96 655	51%	46%	51%	56%	
Quintile of IMD2015 income domain						<0.001
Least deprived	40 630	21%	1%	17%	54%	
2	42 092	22%	6%	21%	36%	
3	39 012	21%	9%	19%	32%	
4	35 351	19%	7%	19%	36%	
Most deprived	32 547	17%	1%	14%	52%	

*Note: P* values are from Chi-squared tests.

Adjusting for cancer site alone (i.e. without any other case-mix adjustor) reduced the size of between-area variance by 76.9% (table 2). Additionally adjusting for deprivation quintile and gender made little further difference, with cumulative reductions of 78.2 and 78.3%. Additionally adjusting for age marginally increased the between-area variance, so that full adjustment (i.e. for cancer site, deprivation quintile, gender and age) reduced the between-area variance by 77.8% compared with the unadjusted model. Although all case-mix variables were associated with stage at diagnosis (*P* < 0.001), adjusting for the three-patient characteristic variables (deprivation, age, gender) had minimal additional impact on between-area variance beyond adjusting for cancer site alone (Supplementary table A2).

**Table 2:** Summary of observed and modelled organizational (Clinical Commissioning Group) performance with and without case-mix adjustment (CMA), with comparisons between results with ‘no CMA’ and those ‘with CMA’

	No case-mix adjustment	Adjusted for cancer site only	Deprivation and cancer site	Gender, deprivation and cancer site	Age group, gender, deprivation and cancer site
Observed 5th–95th percentile range of CCG proportion of cases diagnosed at stages I–II (absolute difference)	0.51–0.62 (0.11)	0.53–0.61 (0.08)	0.54–0.61 (0.07)	0.54–0.61 (0.07)	0.54–0.61 (0.07)
Kendall’s tau-b correlation of adjusted CCG early-stage proportions with results obtained when no case-mix adjustment is used	NA	0.6	0.52	0.52	0.53
% of CCGs that change classification in respect of the ‘at least 60% of cases diagnosed in stages I–II’ target compared with when no case-mix adjustment is used	NA	16.9%	17.4%	17.4%	17.9%
Estimated reduction in rescaled variance compared with that obtained when no CMA is used (see also Methods)	NA	76.9%	78.2%	78.3%	77.8%

**Figure 1:**
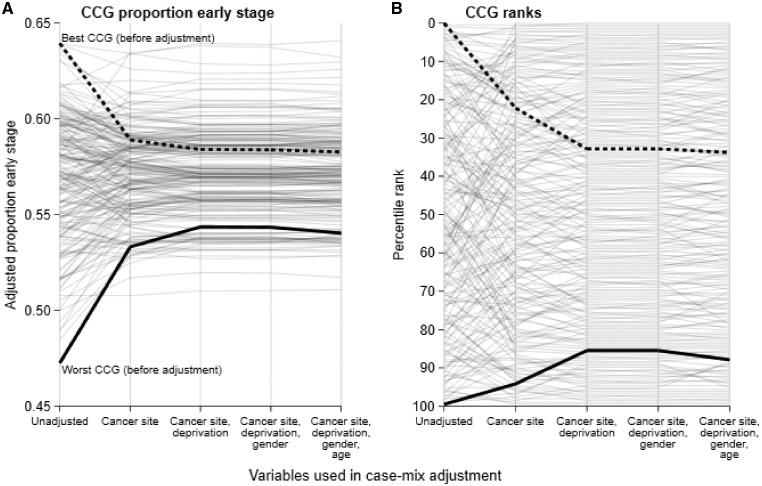
Paired-coordinate plots tracking (A) absolute Clinical Commissioning Group (CCG) performance and (B) CCG rankings across the different specifications of case-mix adjustment (CMA) in order from no adjustment to adjusted for all considered case-mix factors. Each light grey line represents an individual organization (CCG). The thick black dashed line highlights the performance and ranking of the CCG with the highest observed proportion of early stage at diagnosis without adjusting for case mix: after adjustment for cancer site around 20% of CCGs have a higher proportion early stage than that CCG; after adjustment for other case-mix variables around 30% of the compared organizations have a higher proportion early stage than that CCG. The thick black line highlights the performance and ranking of the organizations with the lowest proportion early stage without adjusting for case mix

CMA affected the absolute and relative performance of individual CCG (figure 1). The CCG with the highest unadjusted early-stage proportion appears to have had average performance given its case mix, with between one in five and one in three CCGs having better performance in each of the case-mix-adjusted results (figure 1B). Adjustment for cancer site, and to a lesser extent deprivation, caused noticeable reordering of CCGs (Supplementary figure A2). There was only moderate agreement between CCG ranks on the crude and the (cancer site-deprivation-gender-age) adjusted indicator (Kendall’s tau = 0.53).

**Figure 2:**
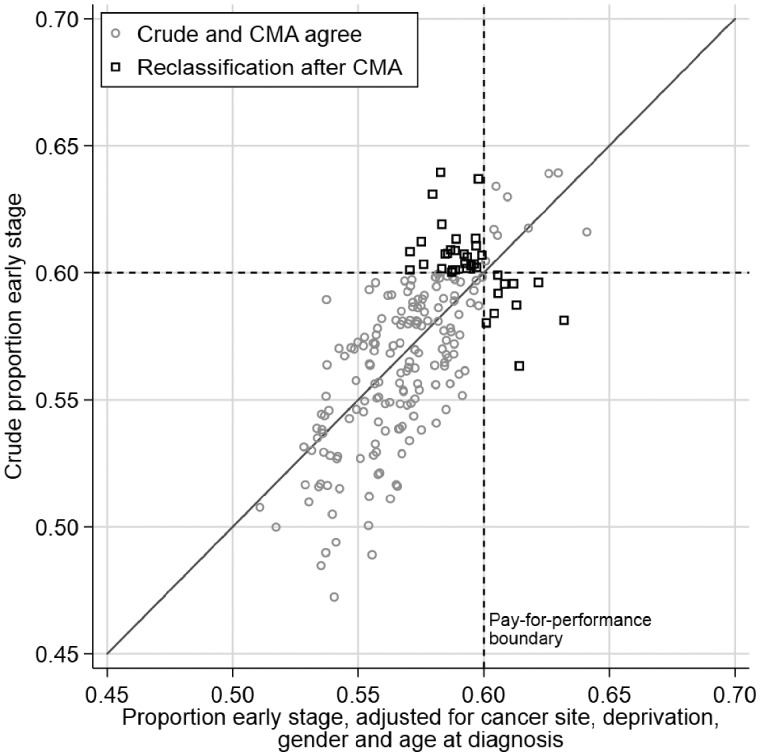
Observed crude proportion of patients diagnosed at an early stage by CCG against case-mix-adjusted proportion of the same organizational indicator, adjusted for cancer site, deprivation, gender and age group at diagnosis. Grey circles represent CCGs (either above or below target proportion of early stage) which remain similarly classified after CMA, whereas black squares represent re-classified CCGs

CMA changed classification on pay-for-performance targets (60% or more of all patients with monitored cancers being diagnosed in stages I–II) for a number of CCGs (figure 2). After CMA, 37 (18%) of all CCGs were reclassified as either meeting or failing the target of at least 60% of their patients being diagnosed at early stage (table 2), 27 (13%) moving from above to below target and 10 (5%) in the opposite direction (figure 2).

## Discussion

Patient case mix, particularly by cancer site and deprivation, has a substantial impact on summary statistics of early stage at diagnosis for geographically defined populations of cancer patients. Adjusting for case mix substantially reduces between-CCG variance and reclassifies about one in six organizations regarding their target attainment status—with several of them moving above or below target.

Guidelines for the production of indicators recommend CMA when the distribution of patient-level factors varies geographically and these factors are strongly associated with the outcome—both conditions being met in our study context.^13–15^ Some public reporting schemes, such as for the Cancer Patient Experience Survey in England, now use synchronous reporting of both crude and case-mix-adjusted scores.^16^ The crude scores show users the actual performance; interpreting them jointly with the adjusted scores helps judge whether an organization attains higher or lower scores than what could be expected by their patient case mix.

Although our study relates to a specific country setting, it has paradigmatic implications for any country with a developed cancer registration system who would consider public health surveillance of stage at diagnosis of cancer, particularly when such indicators combine information from multiple cancer sites.

We used the same national, population-based data as used in early-stage indicators. The large sample size allows for adequately precise estimation of the impact of CMA. Additional adjustors could be considered, including ethnicity, comorbidity and tumour morphology, but their contribution above and beyond that of the four adjustors we examined is likely to be small.

There is debate about whether adjustment for measures of socioeconomic status is justifiable. Such adjustment can be viewed as ‘accepting’ of worse outcomes in patients from more deprived communities, yet lack of adjustment produces performance indicators that reflect broader socioeconomic factors than organizational performance.^17–19^ In the context of our study, this debate is largely redundant, as adjustment for cancer site greatly dominates the degree of changes that result from additional adjustments (including for deprivation, see Results, table 2 and Supplementary Appendices 1 and 2).

The motivating principle for our inquiry is the need for better understanding, and better description to users, of the technical and statistical properties of indicators in current use. The early-stage indicator examined in our study is a summary statistic considering multiple cancer sites. Summary indicators generally give more precise estimates but can mask variation between sub-populations. For example, one CCG may have a lower than average proportion of advanced stage for patients with lung and a higher than average proportion for breast cancer, and another CCG a higher than average proportion for lung and lower than average for breast, but these genuine differences may average out so that the two CCGs have the same performance on the early-stage indicator. Summary indicators may alert users to broad failings in certain organizations, but have the potential to mask heterogeneity and failings in specific domains. Identifying the full nature of such potential problems and guiding improvement actions requires further detailed enquiry.

Our results demonstrate that the case mix of diagnosed patients explains much of the observed variance in area statistics for stage at diagnosis. Informative reporting of such early-stage indicators for geographically defined populations must account for patient case mix, particularly regarding cancer site. Taken together with prior evidence, the findings indicate that early-stage indicators need to account for potential bias from missing data, chance variation and patient case mix.^3^ Direct standardization for cancer site alone (e.g. using the distribution of cancer sites among the nationwide cohort of incident patients) would be an easily implementable solution to reduce bias due to both random and systematic differences in case mix in the early-stage indicator, and would provide for a more appropriate public health and health system performance measure. We recommend the introduction of synchronous reporting of both observed (crude) and adjusted proportions of early stage at diagnosis to support the interpretation of such indicators and enable fair organizational comparisons.

## Supplementary Material

ckz024_Supplementary_Data
