# Supplementary material for: The influence of patient case mix on public health area statistics for cancer stage at diagnosis: a cross-sectional study
Source: Eur J Public Health. 2019 Mar 14;29(6):1103–7. doi: 10.1093/eurpub/ckz024 (PMC6896974; doi:10.1093/eurpub/ckz024)

**APPENDIX 1. Comparisons between observed CCG scores and ranks under different case-mix adjustment models**

**Appendix 1 Table 1. Stage at diagnosis by cancer site, England 2015**

| **Cancer site** |  | **Total diagnoses** |  | **Diagnoses with missing stage** | |  | **Staged diagnoses** |  | **TNM stage** | | | | | | | | | | |
| --- | --- | --- | --- | --- | --- | --- | --- | --- | --- | --- | --- | --- | --- | --- | --- | --- | --- | --- | --- |
|  |  |  |  |  |  |  |  |  | **I** | |  | **II** | |  | **III** | |  | **IV** | |
|  |  | **N** |  | **N** | **(% of total)** |  | **N** |  | **N** | **(% of staged)** |  | **N** | **(% of staged)** |  | **N** | **(% of staged)** |  | **N** | **(% of staged)** |
| Colon |  | 22,903 |  | 2,342 | (10%) |  | 20,561 |  | 3,158 | (15%) |  | 6,081 | (30%) |  | 5,648 | (27%) |  | 5,674 | (28%) |
| Rectum |  | 11,055 |  | 910 | (8%) |  | 10,145 |  | 2,407 | (24%) |  | 1,969 | (19%) |  | 3,524 | (35%) |  | 2,245 | (22%) |
| Lung |  | 37,200 |  | 2,781 | (7%) |  | 34,419 |  | 6,194 | (18%) |  | 2,789 | (8%) |  | 7,227 | (21%) |  | 18,209 | (53%) |
| Melanoma |  | 12,655 |  | 859 | (7%) |  | 11,796 |  | 8,184 | (69%) |  | 2,547 | (22%) |  | 768 | (7%) |  | 297 | (3%) |
| Breast |  | 44,877 |  | 3,180 | (7%) |  | 41,697 |  | 18,388 | (44%) |  | 17,079 | (41%) |  | 3,894 | (9%) |  | 2,336 | (6%) |
| Endometrial |  | 7,197 |  | 416 | (6%) |  | 6,781 |  | 4,976 | (73%) |  | 492 | (7%) |  | 793 | (12%) |  | 520 | (8%) |
| Ovarian |  | 4,880 |  | 704 | (14%) |  | 4,176 |  | 942 | (23%) |  | 292 | (7%) |  | 1,851 | (44%) |  | 1,091 | (26%) |
| Prostate |  | 39,608 |  | 3,955 | (10%) |  | 35,653 |  | 12,258 | (34%) |  | 7,912 | (22%) |  | 7,818 | (22%) |  | 7,665 | (21%) |
| Renal |  | 8,653 |  | 1,075 | (12%) |  | 7,578 |  | 3,668 | (48%) |  | 672 | (9%) |  | 1,362 | (18%) |  | 1,876 | (25%) |
| Bladder |  | 8,372 |  | 1,101 | (13%) |  | 7,271 |  | 3,426 | (47%) |  | 2,050 | (28%) |  | 535 | (7%) |  | 1,260 | (17%) |
| NHL |  | 11,186 |  | 1,631 | (15%) |  | 9,555 |  | 1,896 | (20%) |  | 1,370 | (14%) |  | 1,911 | (20%) |  | 4,378 | (46%) |
| *Grand Total* |  | *208,586* |  | *18,954* | *(9%)* |  | *189,632* |  | *65,497* | *(35%)* |  | *43,253* | *(23%)* |  | *35,331* | *(19%)* |  | *45,551* | *(24%)* |

**Appendix 1 Table 2. Kendall’s Tau correlation coefficients between CCG-level crude and case-mix adjusted early stage at diagnosis indicators**

|  | **No CMA** | **Adjusted for cancer site only** | **IMD and cancer site** | **Gender, IMD and cancer site** | **Age group, gender, IMD and cancer site** |
| --- | --- | --- | --- | --- | --- |
| **No CMA** | 1 |  |  |  |  |
| **Adjusted for cancer site only** | 0.60 | 1 |  |  |  |
| **IMD and cancer site** | 0.52 | 0.88 | 1 |  |  |
| **Gender, IMD and cancer site** | 0.52 | 0.88 | 0.99 | 1 |  |
| **Age group, gender, IMD and cancer site** | 0.53 | 0.88 | 0.94 | 0.94 | 1 |

**Appendix 1 Figure 1. Matrix plot of CCG proportion early stage at diagnosis under crude and various case-mix adjusted early stage at diagnosis indicators**

**
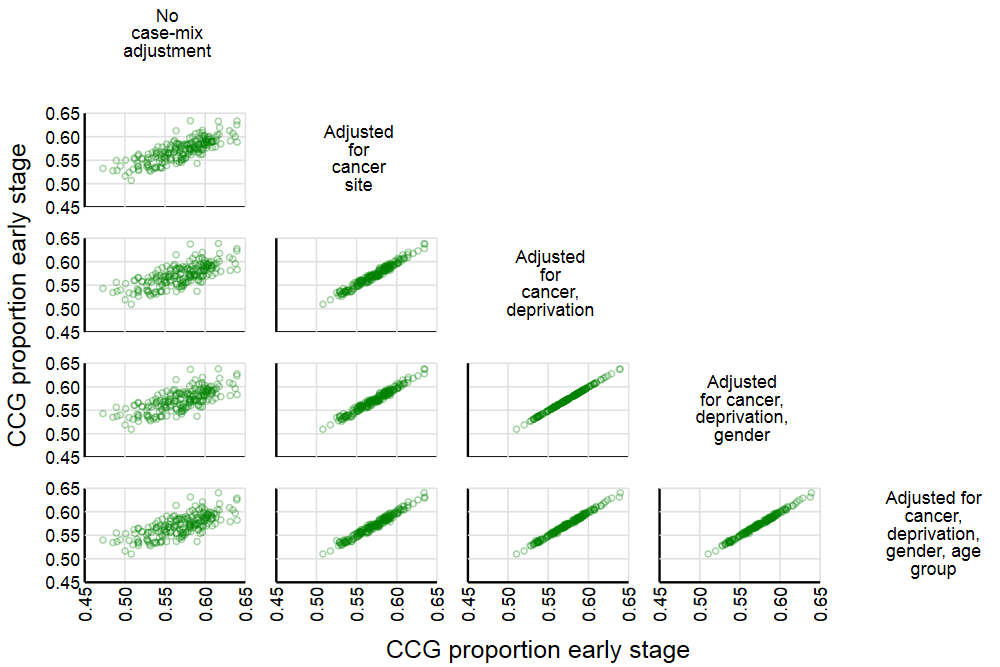
**

**Appendix 1 Figure 2. Matrix plot of CCG ranks under crude and various case-mix adjusted early stage at diagnosis indicators**


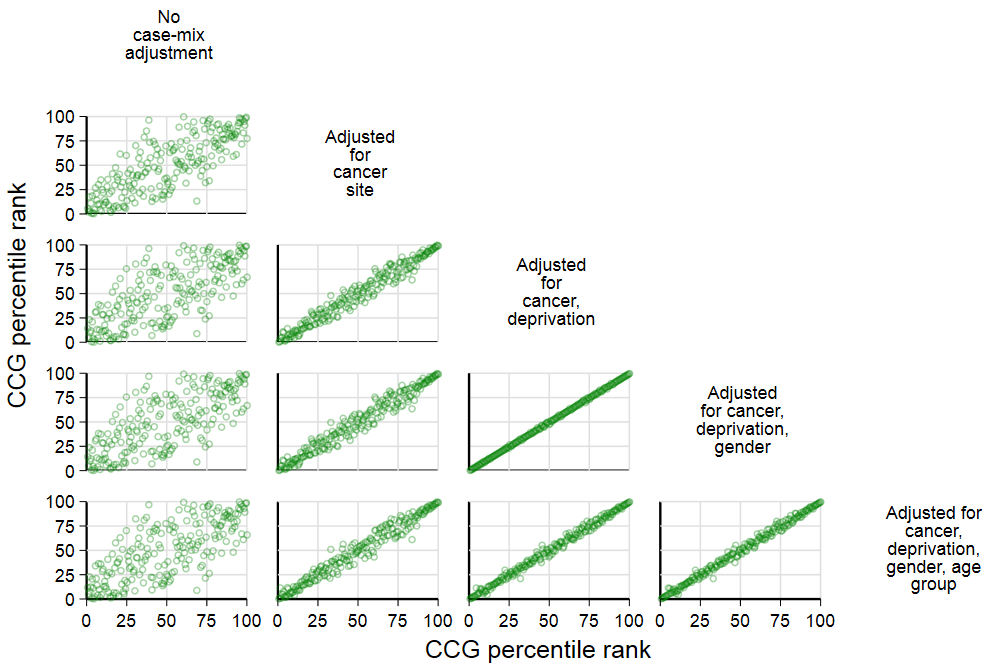

Supplement: ckz024_Supplementary_Data [file ckz024_supplementary_data.docx]
